# Supplementary material for: Progressive External Ophthalmoplegia in Polish Patients—From Clinical Evaluation to Genetic Confirmation
Source: Genes (Basel). 2020 Dec 31;12(1):54. doi: 10.3390/genes12010054 (PMC7824435; doi:10.3390/genes12010054)
Supplement: Supplementary file 1 [file genes-12-00054-s001.zip › Supplementary/Supplementary materials.docx]

**Supplementary materials**

**Long 16kb PCR** was performed using primers mt16426F and 16425R (Zhang et al., 2012) and the Expand Long Template PCR System (Roche). PCR was carried out in 25 µl volume consisting of 2.5 µl 10x PCR buffer number 3, 0.5 mM of each dNTP, 0.95 U polymerase, 0.07 µM of each primer and 100 ng of DNA template. PCR was performed at 92ºC for 2 min, 10 cycles at 92ºC for 10s, 55ºC for 30s, 68ºC for 15 min and 25 cycles at 92ºC for 15s, 55ºC for 30s, 68ºC for 15 min + 20s per cycle and final elongation at 68ºC for 20 min.

***POLG2*** was amplified in the form of 9 PCR products. Reactions were performed in 25 µl final volume containing 2.5 µl 10x PCR buffer, 0.16 µM of each of the forward and reverse primer, 0.4 mM of dNTPs, 1 U of Taq polymerase (A&A Biotechnology) and 100 ng of DNA. PCR conditions were 95°C for 3 min, followed by 35 cycles of 94°C for 1 min, appropriate annealing temperature (56°C for POLG2-2, 3, 7 and 8, 60ºC for POLG2-1a, 1b, 4 and 5 and 64°C for POLG2-6) for 1 min, 72°C for 1.5 min and final elongation at 72°C for 5 min.

***TWNK*** was amplified in form of 6 PCR products. Reactions were performed in 25 µl final volume containing 0.16 µM of each of the forward and reverse primer, 1x concentrated PCR Mix (A&A Biotechnology) and 100 ng of DNA. PCR conditions were 94°C for 3 min, followed by 35 cycles of 94°C for 30s, 58ºC for 30s, 72°C for 1 min and final elongation at 72°C for 7 min.

***SLC25A4*** was amplified in form of 4 PCR products. Reactions were performed in 25 µl final volume containing 0.16 µM of each of the forward and reverse primer, 1x concentrated PCR Mix (A&A Biotechnology), 1% of DMSO for SLC-1 and 100 ng of DNA. PCR conditions were 94°C for 3 min, followed by 35 cycles of 94°C for 30s, 58ºC for 30s, 72°C for 1 min and final elongation at 72°C for 7 min.

***TK2*** was amplified in form of 10 PCR products. Reactions were performed in 25 µl final volume containing 2.5 µl 10x PCR buffer, 0.16 µM of each of the forward and reverse primer, 0.4 mM of dNTPs, 1 U of Taq polymerase (A&A Biotechnology) and 100 ng of DNA. PCR conditions were 94°C for 4 min, followed by 35 cycles of 94°C for 30s, appropriate annealing temperature (58°C for TK2-2, 6, 7 and 9 or 60ºC for TK2-1, 3, 4, 5, 8 and 10) for 30s, 72°C for 1 min and final elongation at 72°C for 5 min.

**Table S1.** Primer sequences.

| gene | primers | primer sequence 5'-3' | exon |
| --- | --- | --- | --- |
| *POLG2* | POLG2-1a | F: TAGCAGAGAACCATCCGAGC | 1 |
|  |  | R: ACTCAGAAGAGAATCCCGGC |  |
|  | POLG2-1b | F: AAGGAGGGCATGTGAAGTCG |  |
|  |  | R: AAGATCCAGCAAGACTGCCT |  |
|  | POLG2-2 | F: AAGTGCTGGGATTATAGGCG | 2 |
|  |  | R: CACTGGTTTGAAGTTCTCGG |  |
|  | POLG2-3 | F: ACAAGAGGCTACCTTATGGC | 3 |
|  |  | R: AACAAACACATCTGAGCCCA |  |
|  | POLG2-4 | F: TGGGCAACAGAATGAGACAC | 4 |
|  |  | R: TGTAGGTGAGTGTCTCTGCA |  |
|  | POLG2-5 | F: AATATGTACTCAGGAGGCTG | 5 |
|  |  | R: GGGATGCCTTGGTTCTATTCT |  |
|  | POLG2-6 | F: AAGGAATCCACCTGCCTCAG | 6 |
|  |  | R: AGGGTTAGGTTGAGCATCCC |  |
|  | POLG2-7 | F: TTGAAGAGGGTGATTTGTGG | 7 |
|  |  | R: TGTGCTCAAACATACTTGCT |  |
|  | POLG2-8 | F: CCCAAATGATGTGTTGAGGT | 8 |
|  |  | R: CTGAATGAAAGCAAGCACCA |  |
| *TWNK* | TW-1ab | F: TGAAGGCACGCTAACCAGGC | 1 |
|  |  | R: AGCATCTCGACGACTAATCA |  |
|  | TW-1cd | F: GCTCGCAGTCTTGTCTTCCC |  |
|  |  | R: GTCGTCTTTCCACTGCCTGT |  |
|  | TW-2 | F: AGCCCTGACCTATGTCTTGG | 2 |
|  |  | R: CCCTGCCCTCTCATTCTTTG |  |
|  | TW-3 | F: ATAATAGAAGGGCAGAGGA | 3 |
|  |  | R: AGACACAGAAGGACAAGAAC |  |
|  | TW-4 | F: ATGGCAGCAGGATGTATGGA | 4 |
|  |  | R: GGATGGACAGTCAAGACGAT |  |
|  | TW-5 | F: CCATTCTTATCACTCCTCCC | 5 |
|  |  | R: GCTCCAGCCTATCCTATCA |  |
| *SLC25A4* | SLC-1 | F: GGCTCGGCGGGACAGATAAC | 1 |
|  |  | R: TCTACGCAGAGGGCACCTTC |  |
|  | SLC-2 | F: TCTCCTGTCCTCTTCCCTTC | 2 |
|  |  | R: TTTCCACACCACCCTCCTTC |  |
|  | SLC-3 | F: CTGAATGAGGAGGTGATGTG | 3 |
|  |  | R: TAGGAGGGAAGCAAGAGTTC |  |
|  | SLC-4 | F: AGGAATGACAGGAGACCCAG | 4 |
|  |  | R: GCTTGGCTGCTGACTGATAC |  |
| *TK2* | TK2-1 | F: CGCCGACTCGCACAAGAAGG | 1 |
|  |  | R: TCCCAGAACCAAAGCCGAGC |  |
|  | TK2-2 | F: GCCAGGGAGTGAGCATAAAC | 2 |
|  |  | R: GGGTGACAGACTTCCTTCTC |  |
|  | TK2-3 | F: CATTATTCCCTGACATTCCC | 3 |
|  |  | R: AAATTACACCTGTGGCTTGC |  |
|  | TK2-4 | F: CAGGGTTCAGCACAGAGAAA | 4 |
|  |  | R: AGGCAGAGGCACCATCATTC |  |
|  | TK2-5 | F: GGTTTCTTGAGCTGTCCTTC | 5 |
|  |  | R: CTTCCTGGCAATCACATACC |  |
|  | TK2-6 | F: CTGCCGCCTTGATTCTCATA | 6 |
|  |  | R: GAGGATTCGTGGCTGTTTGT |  |
|  | TK2-7 | F: GGAATTGCATAGCCCAGAAG | 7 |
|  |  | R: CCCAGGAGAGAGACAAGAGA |  |
|  | TK2-8 | F: TGTGTGCCTGCTTTGCTTCC | 8 |
|  |  | R: CAGAGGTGGTTTCCCAGTTT |  |
|  | TK2-9 | F: TCTGCTTGACACCCTTGGTA | 9 |
|  |  | R: CTTCCCACCTTCCTTCTTCT |  |
|  | TK2-10 | F: TGAAAGGAGGATGCTGCTGA | 10 |
|  |  | R: GAGACGCATGACAAAGACAC |  |

**Table S2.** Full presentation of genetic analysis.

Table in pdf/Excel format.

**Abbreviations:** M – male, F – female, nd – not done / no data, “-“ – negative result, “+” – positive result
